# A Combined X-ray Absorption and UV–Vis Spectroscopic Study of the Iron-Catalyzed Belousov–Zhabotinsky Reaction

**DOI:** 10.1021/acs.jpclett.4c03490

**Published:** 2025-02-14

**Authors:** Giorgio Capocasa, Marika Di Berto Mancini, Federico Frateloreto, Daniele Del Giudice, Osvaldo Lanzalunga, Stefano Di Stefano, Paola D’Angelo, Francesco Tavani

**Affiliations:** Dipartimento di Chimica, Universitá degli Studi di Roma La Sapienza, P.le A. Moro 5, I-00185 Rome, Italy

## Abstract

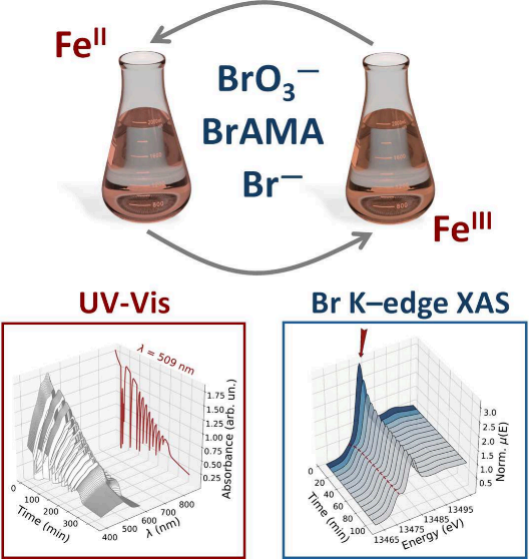

The iron-catalyzed Belousov–Zhabotinsky (BZ) oscillating reaction was investigated in an unstirred reactor by combining Br K-edge X-ray absorption and UV–vis spectroscopies. The experimental data were analyzed through an integrated approach based on principal component analysis, multivariate curve resolution, and *ab initio* theoretical X-ray absorption spectroscopy (XAS), providing quantitative insights into the properties of the key reaction bromine species while contextually tracking the Fe^2+^ to Fe^3+^ oscillatory transformation. The high-quality XAS experimental data supported by the multivariate and theoretical analyses provide clear-cut evidence of the conversion of bromate, initially predominant in the reaction mixture, to the brominated derivative of the employed allylmalonic acid substrate. The described interdisciplinary method was proven to be valuable to monitor the fate of the main BZ reaction brominated species, which are silent to conventional spectroscopic methods of detection, and the developed approach may support future mechanistic investigations of other oscillatory systems.

The Belousov–Zhabotinsky (BZ) oscillating reaction is one of a class of fascinating chemical phenomena^1,2^ which results in the establishment of a nonlinear chemical oscillator kept far from the thermodynamic equilibrium.^1,3−5^ Significant efforts have been devoted to investigating the mechanisms of BZ systems^1,3,4,6−22^ as well as to exploiting BZ oscillatory properties for practical applications, such as for the development of self-swelling gels^23,24^ and chemical computing devices.^25−27^ Since the discovery of the classical BZ oscillating reaction, which is based on the cerium-catalyzed bromate oxidation of citric acid,^1^ numerous oscillating reactions involving other dicarboxylic acids (such as malonic acid, malic acid, and their derivatives) and catalyzed by different metals such as iron, ruthenium, copper, and manganese have been described.^28−33^ The essential mechanism to explain the oscillations of metal-catalyzed BZ reactions was proposed by Field, Köros. and Noyes (FKN)^9^ and involves (i) bromide consumption, (ii) autocatalytic formation of the BrO_2_^•^ radical as the oxidizing intermediate and oxidation of the metal catalyst, and (iii) reduction of the metal catalyst through the organic substrate oxidation which leads to new bromide releases.^33^ However, when a BZ system is left unstirred, its dynamics becomes more complex as it will not only be regulated by the (local) reaction kinetics but also by diffusion and convection effects.^34,35^ Indeed, chaotic behavior has also been observed in unstirred BZ reactions^36^ and chaotic transient phases have been reported to occur between periodic phases during cerium- and iron-catalyzed BZ processes.^37−39^ A key common element in both stirred and unstirred BZ oscillators is the inclusion of bromine, which shuttles between different oxidation states during the BZ reactive cycles. However, tracking the fate of the main BZ brominated species in real time remains a great challenge since bromine chemistry is largely silent to conventional spectroscopic methods.^40,41^

In order to achieve a step forward in the understanding of BZ oscillatory processes, one may turn to X-ray absorption spectroscopy (XAS). XAS is an element-selective technique highly sensitive to the electronic and structural properties of the selected photoabsorber species, and it has been proven valuable in gaining mechanistic insights into reactions occurring in the millisecond to hour time scales.^42−44^ Moreover, the combined use of XAS with a complementary experimental spectroscopic probe such as UV–vis to follow a chemical reaction in real time may provide valuable information that cannot be accessed by either of the two techniques alone. We have recently employed Br K-edge XAS to track the evolution of the brominated species during the unstirred classic cerium-catalyzed BZ reaction carried out in the presence of a relatively high cerium concentration ([Ce_tot_] ≈ 34 mM),^45^ evidencing the occurrence of collective oscillations of the concentrations of the main brominated reaction compounds. Here, we focused our attention on the unstirred iron-based BZ reaction performed in the presence of catalytic Fe^2+/3+^ amounts ([Fe_tot_] = 3 mM). In particular, we employed the XAS technique to monitor the evolution of the key brominated BZ species while contextually following the Fe^2+^-to-Fe^3+^ oscillatory transformation by means of UV–vis spectroscopy. Moreover, we applied an approach based on principal component analysis (PCA), multivariate curve resolution (PCA), and theoretical XAS simulations to determine the number, nature, concentration time evolution, and structure of the reaction key brominated components.

Our study began by monitoring an iron-catalyzed BZ reaction by means of UV–vis and time-resolved XAS, separately. Ferroine (3.0 mM), H_2_SO_4_ (0.48 M), NaBrO_3_ (80 mM), KBr (8.0 mM), and allylmalonic acid (AMA, 50 mM) were first mixed in aqueous solution at 25 °C in an unstirred cell with height, inner width, and inner depth dimensions of 52.0 mm, 9.5 mm, and 1.0 mm, respectively (see Figure S1 in the Supporting Information). Under these experimental conditions oscillations in the concentrations of the Fe^2+/3+^ ions arise, as shown in Figure 1b, in line with previous experimental work on the iron-catalyzed BZ reaction.^39,46^ In particular, this oscillatory behavior is evidenced by the UV–vis kinetic trace at λ = 509 nm, where after an induction time period of 7 min,^4^ periodic variations occur. The oscillations are sustained up to ∼250 min from start, and in our experimental conditions, it is expected that the BZ reaction proceeds in general agreement with the FKN model, with the AMA acid and bromoallylmalonic acid (BrAMA) substituting malonic acid (MA) and bromomalonic acid (BrMA), respectively, in steps R8–R10 (see Figure 1a and Table 1).^33,46^

**Figure 1:**
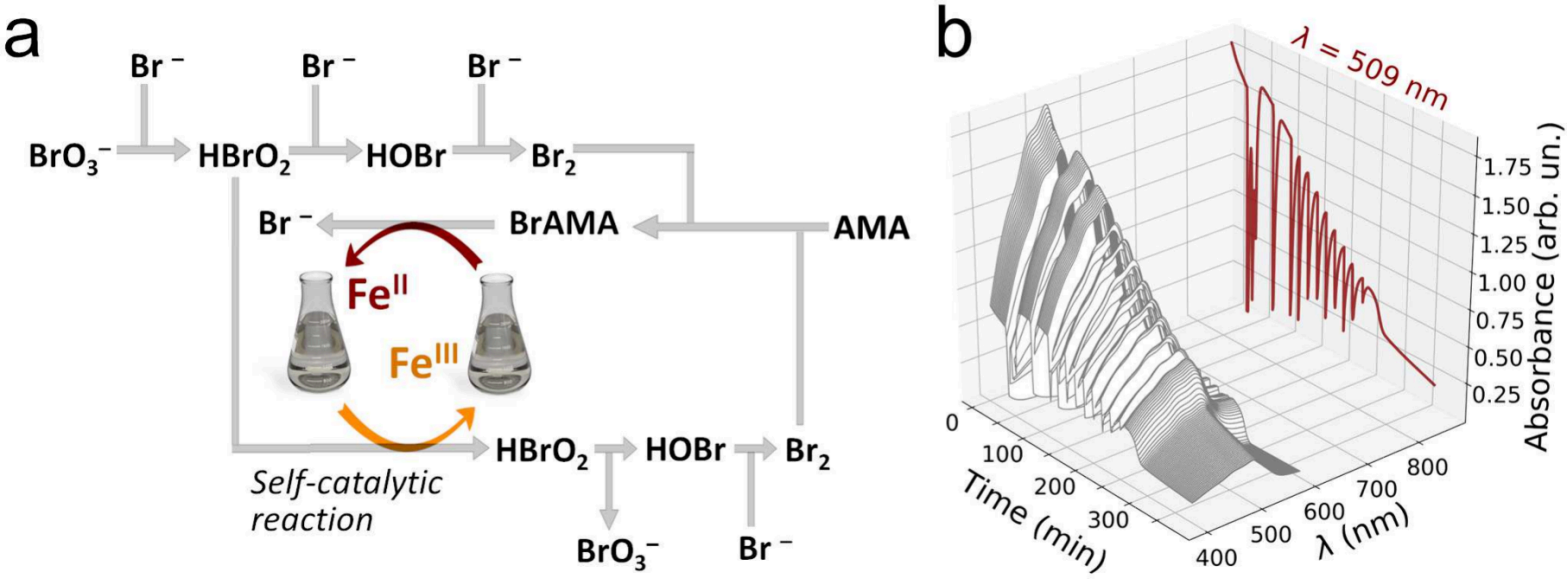
(a) Schematic overview of the FKN reactive processes that take place during the iron-ion-catalyzed BZ reaction. (b) UV–vis monitoring of the BZ reaction involving ferroine (3.0 mM), H_2_SO_4_ (0.48 M), NaBrO_3_ (80 mM), KBr (8.0 mM), and AMA (50 mM). The kinetic trace measured at λ = 509 nm is displayed in dark red behind the time-resolved UV–vis spectra.

**Table 1:** Key Processes Involved in the FKN Mechanism of the Iron-Ion-Catalyzed Belousov–Zhabotinsky Reaction^a^,^33^

Step	Reaction
R1	HOBr + H^+^ + Br^–^*⇌* Br_2_ + H_2_O
R2	HBrO_2_ + H^+^ + Br^–^*⇌* 2HOBr
R3	BrO_3_^–^ + 2H^+^ + Br^–^*⇌* HBrO_2_ + HOBr
R4a	2HBrO_2_*⇌* BrO_3_^–^ + HOBr + H^+^
R4b	HBrO_2_ + H_2_BrO_2_^+^ → BrO_3_^–^ + HOBr + 2 H^+^
R5a	BrO_3_^–^ + HBrO_2_ + H^+^*⇌* Br_2_O_4_ + H_2_O
R5b	Br_2_O_4_*⇌* 2BrO_2_^•^
R6	BrO_2_^•^ + Fe(phen)_3_^2+^ + H^+^ *⇌* HBrO_2_ + Fe(phen)_3_^3+^
R8a	Br_2_ + AMA(enol) → BrAMA + Br^–^ + H^+^
R8b	HOBr + AMA(enol) → BrAMA + H_2_O
R9	Fe(phen)_3_^3+^ + BrAMA *⇌* P1 + Fe(phen)_3_^2+^ + Br^–^ + 2H^+^
R10	Fe(phen)_3_^3+^ + AMA → P2 + H^+^ + Fe(phen)_3_^2+^
E	AMA *⇌* AMA(enol)
A1	H_2_BrO_2_^+^*⇌* HBrO_2_ + H^+^

a AMA = allylmalonic acid; BrAMA = bromoallylmalonic acid.

We then turned to XAS to gain new information about the structural and electronic modifications of the Br-related species during the BZ process. To this end, ferroine (3.0 mM), H_2_SO_4_ (0.48 M), NaBrO_3_ (80 mM), KBr (8.0 mM), and AMA (50 mM) were mixed in aqueous solution at 25 °C in a cell with height, inner width, and inner depth dimensions equivalent to those of the cell employed during the UV–vis experiment. The ensuing unstirred BZ reaction was then followed by time-resolved X-ray near edge structure (XANES) spectroscopy, and the collected XANES spectra are shown in Figure 2. Looking at the XANES spectra measured up to 106.3 min from start (Figure 2b), a clear evolution of the main spectral features is observed. In particular, the intensities of both the pre-edge peak at 13472.5 eV and white line transition at 13477.7 eV transitions tend to increase and decrease, respectively, as a function of time. The entire XANES data set recorded during the BZ reaction is displayed in Figure 2a, where the first measured XAS spectrum (*t* = 3.7 min) is shown in red and the XAS spectrum collected at *t* = 806.7 min is displayed in blue. Note that during this time period, an intense pre-edge feature at 13472.5 eV arises, suggesting the formation of a new brominated species.

**Figure 2:**
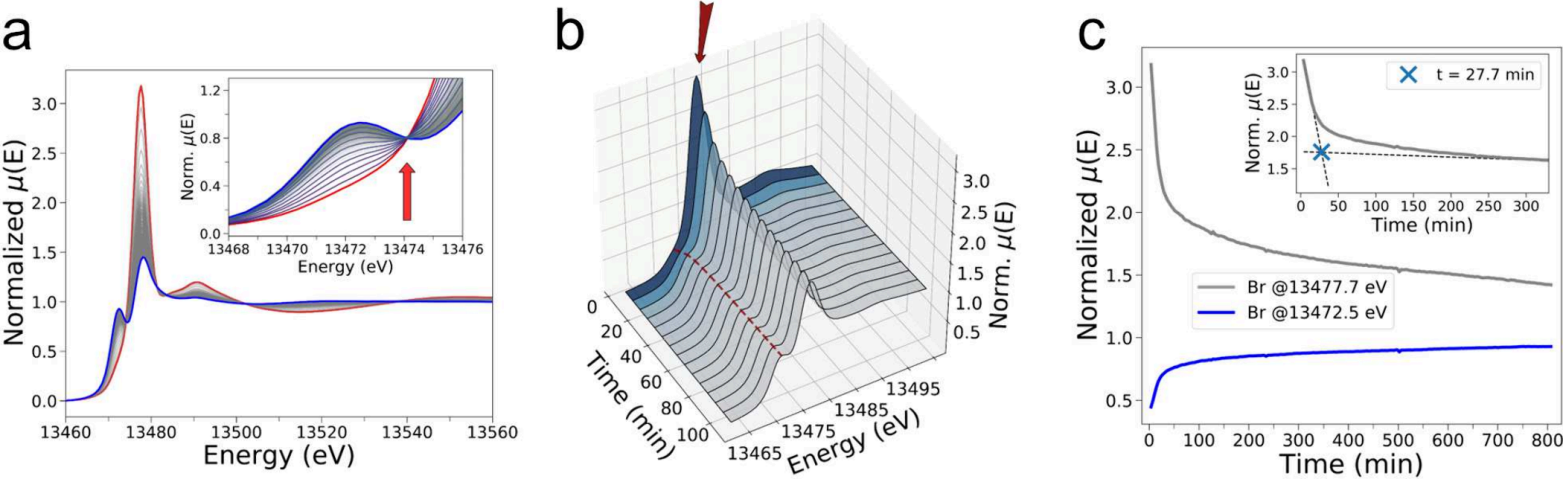
Time evolution of the Br K-edge XANES spectra of the BZ reaction involving ferroine (3.0 mM), H_2_SO_4_ (0.48 M), NaBrO_3_ (80 mM), KBr (8.0 mM), and AMA (50 mM). (a) 2D representation of the Br K-edge XANES spectra. The inset shows an enlargement of the pre-edge region, while a red arrow indicates the presence of an isosbestic point at 13474.1 eV. (b) 3D depiction of the Br K-edge XANES spectra with a dotted line and dark red arrow highlighting the transitions located at 13472.5 eV and at 13477.7 eV, respectively. (c) Intensity variation at 13472.5 eV (blue line) and at 13477.7 eV (gray line) of the time-resolved Br K-edge XAS spectra measured during the investigated iron-catalyzed BZ reaction. The inset presents a magnification of the intensity evolution at 13477.7 eV, where the intersection of extrapolated dashed lines approximates the time (*t* = 27.7 min) after which the XAS intensity undergoes a slower decay.

The evolution of the XAS intensities measured at 13472.5 and 13477.7 eV is displayed in Figure 2c. Looking at this figure, one may observe that the pre-edge and white line intensities increase and decrease in a smooth, opposite manner during the BZ process. Further, the decay of the intensity measured at 13477.7 eV and the increase of that at 13472.5 eV are more rapid during the reaction initial phase that may be estimated to last up to 27.7 min from reaction start (see inset of Figure 2c) and then progressively slow down as the reaction proceeds. Importantly, the first collected XAS spectrum (red curve in Figure 2a) strongly resembles that of a 0.1 M aqueous solution of NaBrO_3_ (the two spectra are compared in Figure S2), in agreement with the fact that BrO_3_^–^ is expected to be the prevalent brominated species in the solution at short reaction times. Moreover, the presence of different isosbestic points in the XAS data (Figure 2a) suggests that the time-resolved XAS data capture the progressive interconversion of BrO_3_^–^ into one prevalent brominated compound.

In order to obtain quantitative insights into the distinct spectral components contributing to the experimental XANES data, we performed a PCA analysis of the time-resolved spectra.^47,48^ The aim of PCA is to reduce the dimensionality of complex data sets by identifying the smallest number of independent components, termed principal components (PCs), whose linear combination best explains the variance of all the variables in the original data set.^43,49,50^ Accordingly, the number of PCs necessary to explain the variance in our own XANES data may be associated with the number of brominated “pure” chemical species contributing to the experimental spectra as the BZ reaction evolves, in agreement with the Lambert–Beer law.^43,44,44,51^ We therefore first performed a scree plot statistical test^45^ to identify the number (*N*) of PCs contributing to the time-resolved XANES data and found that *N* = 3, as shown in Figure 3a. Indeed, the presence of an elbow in the scree plot supports the notion that, for a number of PCs greater than 3, the associated singular values are attributed to statistical noise.^47,48^ Consistently, the percentage residual error committed in reconstructing the XAS data set with *N* = 3 is negligible, never exceeding a value of 0.006% in the reconstruction of individual XAS spectra (Figure S3c), and experimental XAS spectra at selected times from reaction start are well reproduced by employing 3 PCs, as shown in Figure 4.

**Figure 3:**
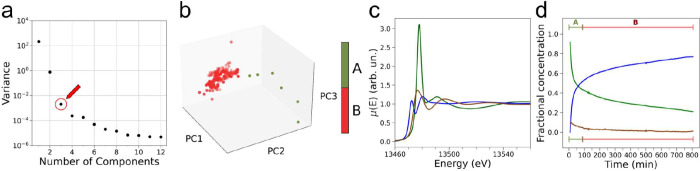
Results of PCA and MCR analyses of the time-resolved Br K-edge XAS spectra. (a) Scree plot with a red circle and arrow indicating the singular value related to *N* = 3. (b) Three-dimensional PCA clustering results of the XANES spectra recorded during the BZ reaction and assigned to reaction stages A (red) and B (green). (c) Extracted XANES spectra associated with the spectrum of BrO_3_^–^ (green line), of Br^–^ (brown line), and of the third component (blue line) which is assigned to BrAMA. (d) Extracted concentration profiles associated with the first (green line), second (brown line), and third (blue line) spectral components.

**Figure 4:**
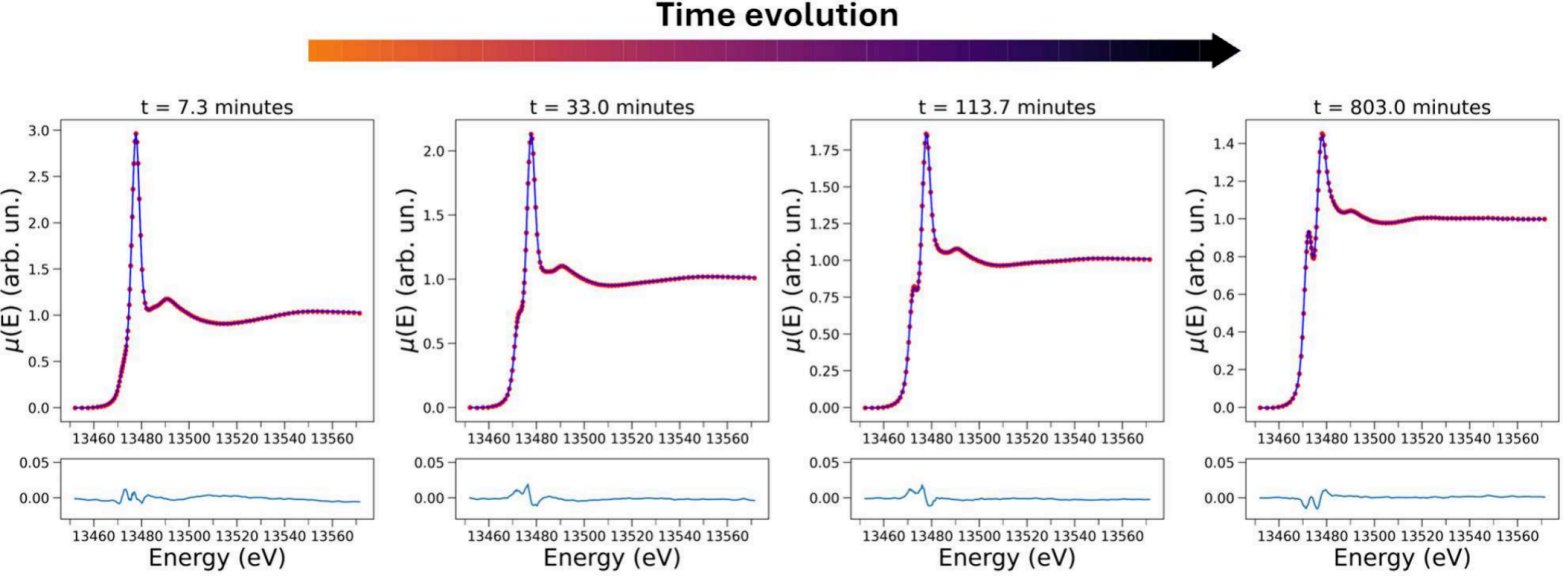
Evolution of experimental XANES spectra (dotted red lines) at selected times from reaction start and of their corresponding reconstructed XANES spectra obtained by employing *N* = 3 PCs (filled blue lines). The error between the given experimental and reconstructed XANES spectrum is displayed in the bottom of each panel.

In the second step of the analysis, the XANES data were analyzed by means of a transition matrix-based MCR approach^52^ using a number of components equal to 3 (full details are reported in Section 2.2 of Supporting Information) in order to gain quantitative information on the nature and concentration time evolution of the key brominated reaction species. The extracted XANES spectra and associated concentration profiles are shown in Figures 3c,d, respectively and were derived by constraining the first and second spectral components to coincide with the XAS spectra of the BrO_3_^–^ and Br^–^ references measured in aqueous solutions.^45^ Looking at Figure 3d, one may observe that the concentrations of BrO_3_^–^ and of the third component decrease and increase at a faster rate up to *t* ≈ 27.7 min, while then they evolve at a slower rate up to *t* = 806.7 min reaching relative abundances of ∼21% and ∼77%, respectively. Further, the concentration of Br^–^ starts from an initial value of ca. 9% (due to the total bromine initially present in the reaction mixture, since [KBr]_0_ = 8.0 mM) and rapidly decays toward values only slightly above zero, as expected due to the initial minority presence of Br^–^ in the reaction mixture and to its consumption during the BZ oscillatory cycles.^33,45^ The concentration trends of BrO_3_^–^ and of the third component exhibit an opposite behavior, since the PCA-assisted analysis identified that two chemical components predominantly contribute to the XANES data. The third XANES spectral component (Figure 3c) exhibits an intense pre-edge transition at 13472.5 eV, in analogy to the XANES spectrum of a 0.1 M methanol solution of diethyl bromomalonate (see Figure S4), a feature that is due to a 1s → 4p dipole-allowed transition in states that result from the covalent bond of the bromine photoabsorber.^53−58^ Importantly, the XANES spectra of the third component and of the diethyl bromomalonate reference exhibit strong similarities, suggesting that the underlying molecular structures of the two brominated compounds also share some degree of similarity. This evidence, together with the fact that according to the FKN model of the Fe-catalyzed BZ reaction (see Table 1) the BrAMA species is the only brominated species whose structure is similar to that of diethyl bromomalonate and that may reasonably accumulate in the reaction mixture, led us to preliminarly assign the third component to BrAMA.

In order to confirm this assignment, an *ab initio* theoretical XAS spectrum was calculated by means of the FDMNES code^59,60^ starting from a DFT-optimized geometrical model of the BrAMA species (the ORCA code^61^ was employed). The associated DFT-optimized structure is shown in Figure 5a, while Figure 5b compares the XANES spectrum of the MCR-extracted third component and the theoretical XANES spectrum. One may observe that the relative energies and intensities of the most intense experimental features **A** → **D** are all correctly reproduced by the XANES calculations, further supporting our identification of the third reaction component as BrAMA. Our assignment is further supported by the fact that the theoretical XANES spectra of HBrO_2_ and HOBr (Figure S5a) and the experimental XANES spectra of Br^–^ and Br_2_ (Figure S5b) display evident differences if compared to the MCR-derived XANES spectrum of the third reaction component. The fact that within our experimental conditions our time-resolved XANES measurements do not detect significant contribution from brominated species other than BrO_3_^–^, Br^–^, and BrAMA, such as Br_2_, HBrO_2_, or HOBr may be explained by the fact that the concentration of the latter species remain well below the method sensitivity during the BZ oscillatory process. We also point out that in the employed experimental BZ conditions, where we use a total iron concentration equal to 3.0 mM, we have not detected any oscillatory behavior in the time-resolved XANES data, contrary to what we recently observed while monitoring the cerium-catalyzed BZ reaction in the presence of a total cerium concentration equal to 34.0 mM.^45^ We attribute this different behavior to the lower metal catalyst concentration employed in this study, as the concentration oscillations of the Fe^2+^/Fe^3+^ couple employed herein are not of sufficient amplitude to lead to appreciable oscillations of the main brominated species.

**Figure 5:**
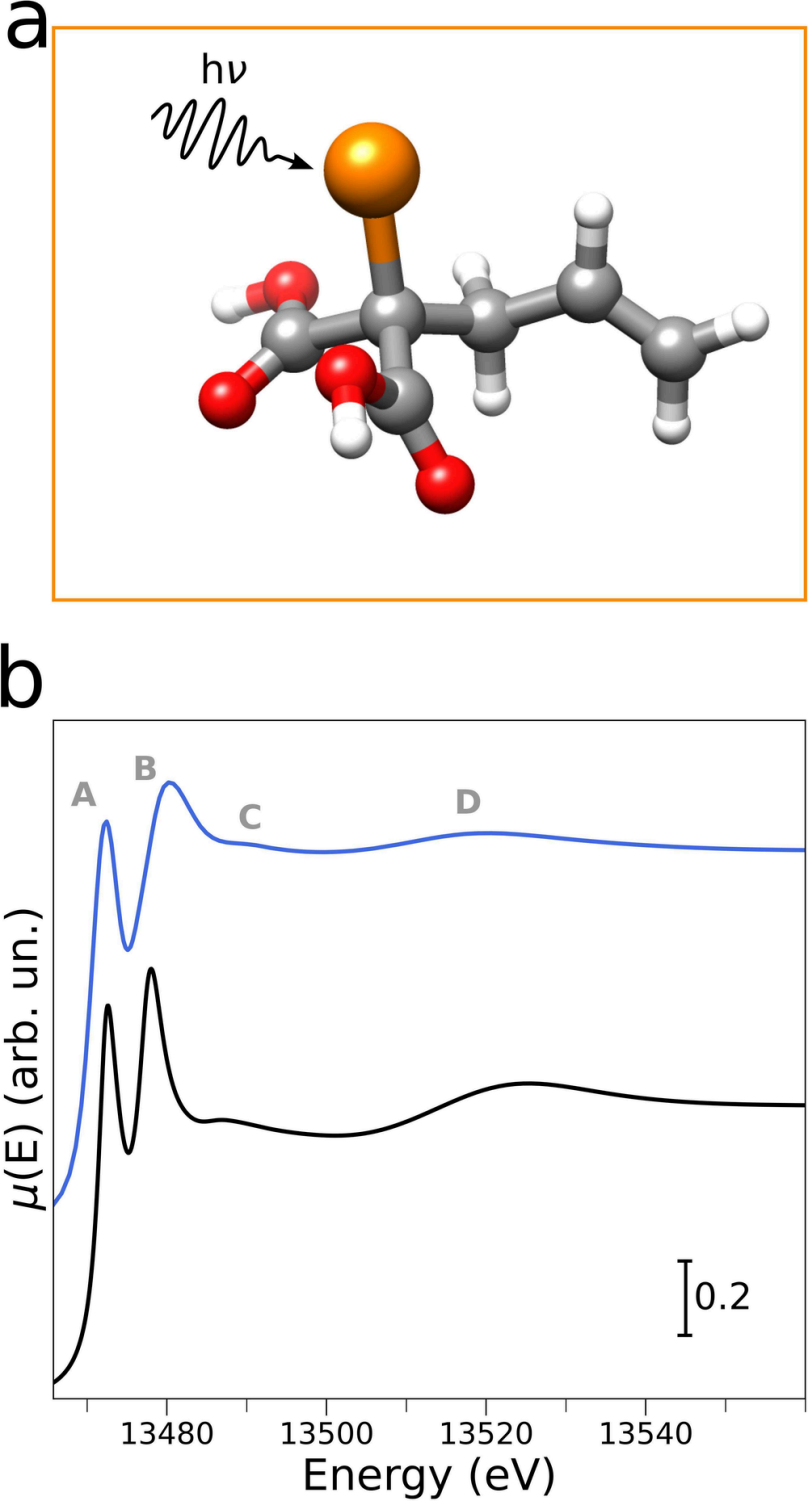
(a) Minimum-energy structure calculated at the DFT/ZORA-def2-TZVP level for the BrAMA species. The bromine, oxygen, carbon, and hydrogen atoms are colored orange, red, black, and white, respectively. (b) Comparison between the experimental and theoretical Br K-edge XANES spectra of BrAMA. The MCR-extracted experimental curve is depicted in light blue, while the simulated one is in black. Letters label the most intense features of the experimental spectrum.

The observed behavior of the BrO_3_^–^ and BrAMA concentrations may be interpreted as follows: Let *v*_3_, *v*_4_, and *v*_5a_ be the forward rates of processes R3, R4a+R4b, and R5a in the FKN mechanism (Table 1), and *v*_8a_, *v*_8b_, and *v*_9_ those of processes R8a, R8b, and R9. By making use of the steady-state approximation,^4^ we can derive the following expressions for the time evolution of the concentrations of BrO_3_^–^ and BrAMA:
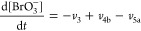
1
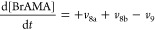
2

According to eq 1, overall the concentration of BrO_3_^–^ decreases during the BZ reaction when *v*_3_ + *v*_5a_ > *v4a+v*_4b_, a condition that we may assume to be true under our experimental conditions. Conversely, the observed concentration evolution of the BrAMA species may be explained by assuming the predominance of processes R8a and R8b over process R9 during the investigated time period of the BZ process, an assumption that leads to *v*_8a_ + *v*_8b_ > *v*_9_ and a gradual buildup of BrAMA content. In turn, the relative concentrations of other brominated species such as Br^–^, Br_2_, HOBr, and HOBr_2_ remain very low and not detectable by the XAS technique. This behavior is consistent with previous experimental and theoretical studies^3,4^ that reported, for instance, Br^–^ concentration oscillations in the order of 1 × 10^–6^ M that is below the sensitivity of our combined XAS-MCR technique.

In conclusion, the combined XAS/UV–vis analysis has been found to be highly effective in providing novel molecular-level insights into the iron-catalyzed BZ reaction. Our innovative approach integrates spectroscopic tools sensitive to both the metal and brominated portions of the BZ system and leverages the capabilities of PCA, MCR, and theoretical XAS spectroscopy to gain a direct, thorough characterization of the main brominated intermediate species arising during the reaction. In particular, the XAS experimental technique has been used to rationalize the number, nature, and concentration time evolution of the main brominated components evolving in the reaction mixture, and the direct transformation of BrO_3_^–^ to BrAMA has been identified while a reduced and negligible accumulation of Br^–^ and other brominated species has been observed, respectively. We stress that obtaining quantitative information on this kind on the speciation of Br-related species is far from trivial due to the fact that many of the brominated BZ reactants and intermediates are silent to conventional spectroscopic methods. On the other hand, the use of UV–vis spectroscopy has proven to be useful in monitoring the oscillatory evolution of the Fe^2+^/Fe^3+^ redox couple. We foresee that our innovative combined experimental and theoretical approach can become a valid method in the toolbox required to monitor BZ and non-BZ oscillatory processes.

## Supporting Information Available

Materials and methods used; theoretical background of the principal component analysis; XANES theoretical calculations; experimental cell picture, XAS spectra of reference compounds; statistical analysis of multivariate reconstruction (PDF)
Transparent Peer Review report available (PDF)
