# Supplementary material for: A Combined X-ray Absorption and UV–Vis Spectroscopic Study of the Iron-Catalyzed Belousov–Zhabotinsky Reaction
Source: J Phys Chem Lett. 2025 Feb 14;16(8):1840–6. doi: 10.1021/acs.jpclett.4c03490 (PMC11873914; doi:10.1021/acs.jpclett.4c03490)
Supplement: Supplementary file 1 — jz4c03490_si_001.pdf [file jz4c03490_si_001.pdf]

Supporting Information

A Combined X-ray Absorption and UV-Vis Spectroscopic Study of the Iron-Catalyzed Belousov-Zhabotinsky Reaction

Giorgio Capocasa, Marika Di Berto Mancini, Federico Frateloreto, Daniele Del Giudice, Osvaldo Lanzalunga,* Stefano Di Stefano,* Paola D'Angelo,* and Francesco Tavani*

Dipartimento di Chimica, UniversitàÂ degli Studi di Roma La Sapienza, P.le A. Moro 5, I-00185 Rome, Italy

E-mail: osvaldo.lanzalunga@uniroma1.it; stefano.distefano@uniroma1.it; p.dangelo@uniroma1.it; francesco.tavani@uniroma1.it

1 Materials

1.1 BZ reaction conditions

The BZ reactions were carried out by mixing ferroine (3.0 mM), H_2SO_4 (0.48 M), $NaBrO_3$ (80 mM), KBr (8.0 mM) and allylmalonic acid (AMA, 50 mM) in aqueous solution at 25 °C. The BZ reactions described in this work were performed in initially homogeneized, unstirred solutions. The reaction cell employed for the UV-Vis independent measurements is shown in Figure S1 and was characterized by height, inner width and inner depth dimensions of 52 mm, 9.5 mm and 1 mm, respectively. Further, the reaction cell employed for the XAS independent measurements was obtained by sealing kapton windows on a cell possessing the same dimensions listed above.

2 Methods

2.1 X-ray Absorption measurements

The Br K-edge XAS spectra of the BZ reaction conducted in the Aluminum alloy cell with the dimensions shown in Figure S1 were collected at room temperature in transmission mode at the Elettra Synchrotron (Trieste, Italy) on the XAFS beamline. The storage ring was operating at 2 GeV with an optimal storage beam current between 300 and 130 mA. The Aluminum alloy cell was sealed using Kapton film windows of 0.5 mm.

2.2 Decomposition of the Br K-edge XANES data into the spectra and fractional concentrations of key components

The XANES spectroscopic measurements yielded a series of spectra that were positioned in a matrix $\boldsymbol{D}$, where the columns of $\boldsymbol{D}$ are the spectra measured at time t. According to Lambert-Beer's law, at any given time a number N of "pure" and independent components

weighed by their fractional concentration contributes to the measured signal[1] and therefore one can decompose the experimental data into the spectra associated to the key species and in their relative concentration profiles. In the present work, such decomposition was performed with the PyFitit code,[1] a software that uses to such end an algorithm belonging to the MCR family.

The starting point is the Singular Value Decomposition (SVD) equation:

$$\boldsymbol{D} = \boldsymbol{U} \cdot \boldsymbol{\Sigma} \cdot \boldsymbol{V} + \boldsymbol{E} \tag{1}$$

where the product $\boldsymbol{U} \cdot \boldsymbol{\Sigma}$ contains, on its N columns, a set of values that may be associated to the normalized absorption coefficients, $\boldsymbol{\Sigma}$ is a diagonal matrix known as the *singular values* term, whose elements are sorted in decreasing order, while $\boldsymbol{V}$ can be interpreted as the concentration matrix associated to the N-selected components. Lastly, the error matrix $\boldsymbol{E}$ represents the lack of fit between the experimental data matrix $\boldsymbol{D}$ and the reconstructed matrix $\boldsymbol{\mu} = \boldsymbol{U} \cdot \boldsymbol{\Sigma} \cdot \boldsymbol{V}$. The SVD deconvolution depends on the correct estimation of the number of components N present in the experimental spectral matrix. To this end, in this investigation we evaluated the percentage error committed in reproducing the experimental data with an increasing number N of components, as detailed in the main text, as shown in Figure S3. The percentage error function has been calculated with the following expression:

$$R(n) = \frac{\sum_{i=1}^{K} \sum_{j=1}^{m} (d_{ij} - \mu_{ij}^{PC=n})^2}{\sum_{i=1}^{K} \sum_{j=1}^{m} (d_{ij})^2} \times 100 \tag{2}$$

where d_{ij} and $\mu_{ij}^{PC=n}$ are the normalized absorbance values for the dataset and for the dataset reconstructed with N = n, respectively (K and m represent the number of acquired spectra and of the energy points, respectively, while $n = 1, 2, ...K$).

At this point, all matrices in Equation 1 are solely mathematical solutions to the decomposition problem without physico-chemical meaning. Once N is established, the approach implemented by PyFitIt requires the introduction of a transformation N $\times$ N matrix $\boldsymbol{T}$ in

Equation 1, using the relation $\boldsymbol{I} = \boldsymbol{T} \cdot \boldsymbol{T}^{-1}$:

$$\boldsymbol{D} = \boldsymbol{U} \cdot \boldsymbol{\Sigma} \cdot \boldsymbol{T} \cdot \boldsymbol{T}^{-1} \cdot \boldsymbol{V} + \boldsymbol{E} \tag{3}$$

where the spectra belonging to the key species are given by $\boldsymbol{S} = \boldsymbol{U} \cdot \boldsymbol{\Sigma} \cdot \boldsymbol{T}$ and their concentration profiles by $\boldsymbol{C} = \boldsymbol{T}^{-1} \cdot \boldsymbol{V}$. Subsequently, the matrix elements T_{ij} of matrix $\boldsymbol{T}$ are modified by sliders to achieve $\boldsymbol{S}$ and $\boldsymbol{C}$ which are chemically and physically interpretable. Once this step is achieved, one can finally write:

$$\boldsymbol{D} = \boldsymbol{S} \cdot \boldsymbol{C} + \boldsymbol{E} \tag{4}$$

The unknown number of T_{ij} elements of $\boldsymbol{T}$ is in principle equal to N^2. In order to reduce such ambiguity, the XANES spectrum of the BrO_3^- and Br^- references were constrained to coincide with the first and second of the three extracted components. This operation allows the reduction of the number of unknown T_{ij} elements from N^2 to $N^2 - N$. In our investigation, a 3×3 matrix $\boldsymbol{T}$ containing nine elements was employed to retrieve the spectral and concentration profiles of the three key species contributing to the time-resolved XANES spectra.

2.3 Determination of the number of principal components through the scree plot statistical test

The Br K-edge spectroscopic data were subjected to the scree plot test in order to assess the number of pure species present in the reaction mixture.

It is possible to demonstrate that:

$$\lambda_i = \frac{\sigma_{ii}^2}{m-1} \tag{5}$$

where σ_{ii} are the singular values extracted by the Singular Value Decomposition procedure and λ_i are the eigenvalues of the covariance matrix of $\mathbf{D}$ (evaluated for m energy steps) relative to every i-th component. These values correspond to the variance of each principal component (PC).[1,2] Consequently, the components with a high σ_{ii} value contribute significantly to the dataset reconstruction, while those with a small σ_{ii} value are associated to noise.

In the scree plot, the singular values relative to each principal component are plotted against the number of PCs. The presence of an elbow in such curve separates the signal and noise related components.

2.4 Theoretical method

Theoretical calculations were performed employing the ORCA code.[4] The geometry of BrAMA was optimized at the Density Functional Theory (DFT) theory level employing the B3LYP functional, the D3BJ dispersion correction, with a ZORA-def2-TZVP basis set. The FDMNES code has been employed to perform the XANES theoretical data analysis using the muffin-tin approximation for the potential and including quadrupole transitions in the calculations.[5,6] FDMNES represents originally a one-electron approach and is based on the ab-initio calculations of the electronic structure and on the resolution of the discretized radial Schrödinger equation. The FDM approach is attractive for the simulation of the photoelectron wave function beyond 100eV above the absorption edge.

3 Supplementary Figures S1–S5

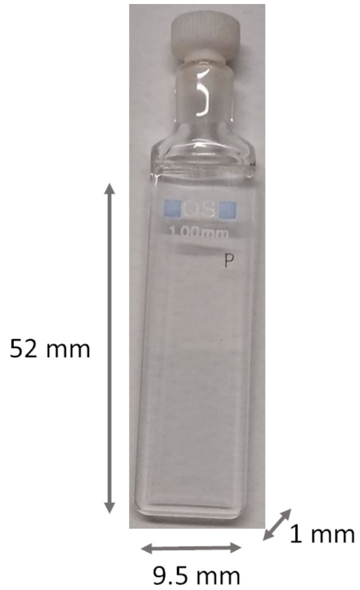

Figure S1: Depiction of the cell employed in this study to monitor the BZ reaction through UV-Vis spectroscopy. The inner dimensions of the cell are listed. An Aluminum alloy cell with same dimensions and Kapton windows was employed to monitor the BZ reaction through XAS.

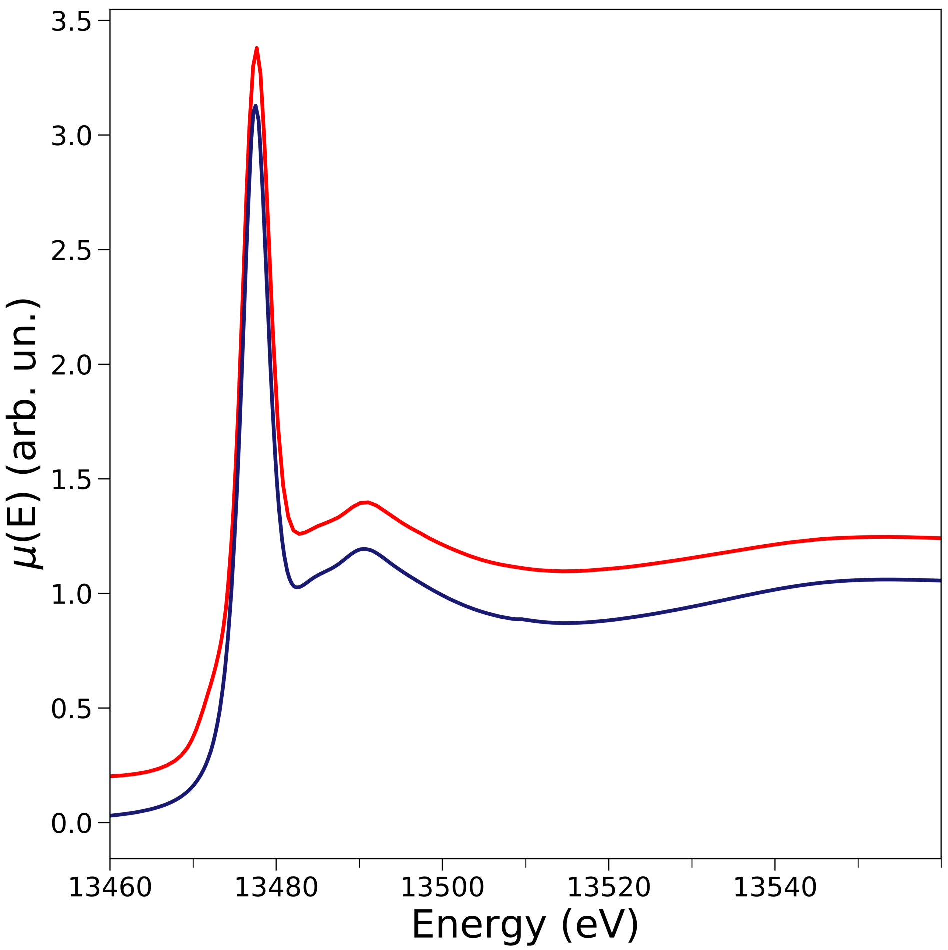

Figure S2: Br K-edge XAS spectra of the investigated BZ reaction measured at $t = 3.33$ min (red line) and of a $NaBrO_3$ 0.1 M (dark blue line) aqueous solution.

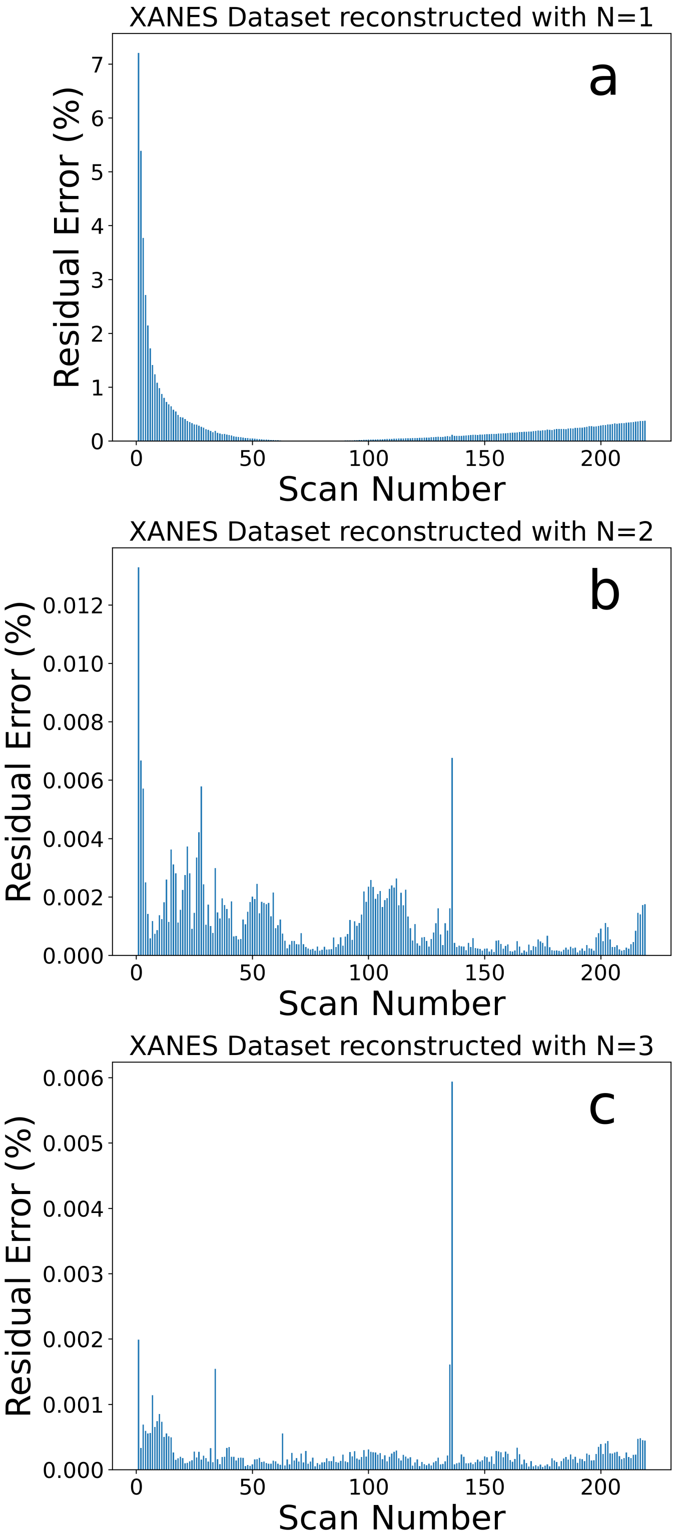

Figure S3: Residual percentage errors related to the reconstruction of the individual XAS spectra while employing N=1 (a), N=3 (b), and N=3 (c) PCs.

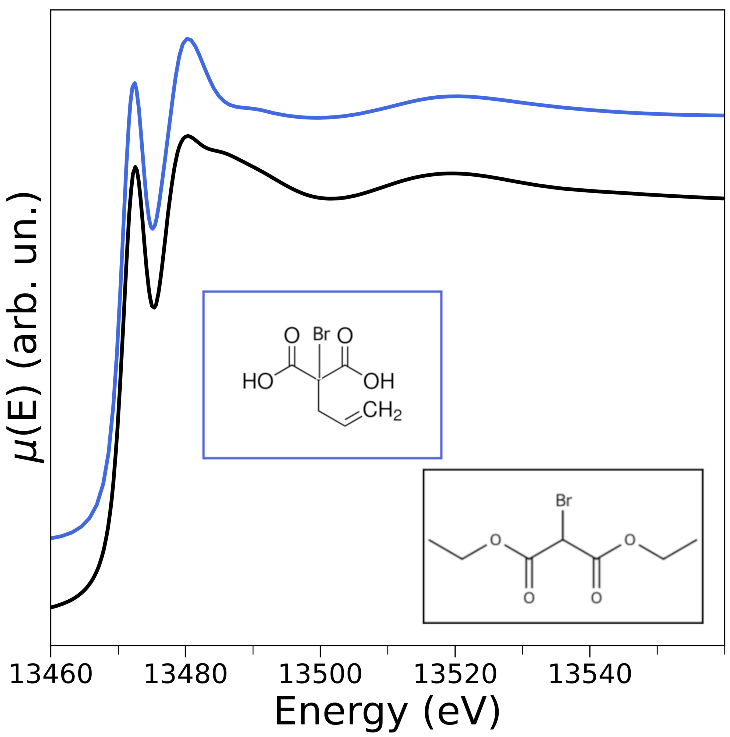

Figure S4: Br K-edge XAS spectra of the reaction intermediate component assigned to the BrAMA species (light blue line) and of the diethyl bromomalonate 0.1 M methanol solution (black line).

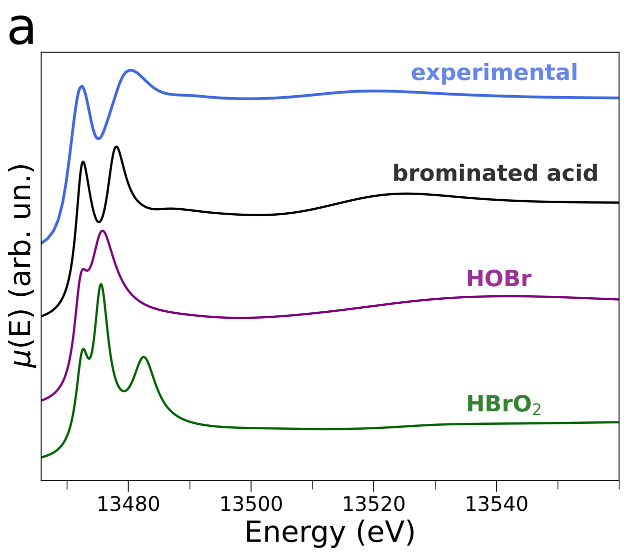

Figure S5: Br K-edge XAS spectrum of the MCR-extracted component compared to the theoretical XAS spectra of different brominated compounds (a) and to the experimental XAS spectra of gaseous Br_2 (orange line) and Br^- in aqueous solution (brown line).

References

(1) Martini, A.; Guda, S.; Guda, A.; Smolentsev, G.; Algasov, A.; Usoltsev, O.; Soldatov, M.; Bugaev, A.; Rusalev, Y.; Lamberti, C.; Soldatov, A. *Comput. Phys. Comm.* **2019**, 107064.

(2) Markovsky, I. *Automatica* **2008**, *44*, 891–909.

(3) Zars, E.; Glaser, R.; Downing, M.; Chicone, C. *J. Phys. Chem. A* **2018**, *122*, 6183–6195.

(4) Neese, F. *Wiley Interdiscip. Rev. Comput. Mol. Sci.* **2012**, *2*, 73–78.

(5) Joly, Y. *Phys Rev B* **2001**, *63*, 125120.

(6) Bunău, O.; Joly, Y. *J. Phys: Condes. Matter* **2009**, *21*, 345501.
