# Supplementary material for: A Combined X-ray Absorption and UV–Vis Spectroscopic Study of the Iron-Catalyzed Belousov–Zhabotinsky Reaction
Source: J Phys Chem Lett. 2025 Feb 14;16(8):1840–6. doi: 10.1021/acs.jpclett.4c03490 (PMC11873914; doi:10.1021/acs.jpclett.4c03490)
Supplement: Supplementary file 2 — jz4c03490_si_002.pdf [file jz4c03490_si_002.pdf]

jz-2024-034903.R1

Name: Peer Review Information for "A Combined X-ray Absorption and UV-Vis Spectroscopic Study of the Iron-Catalyzed Belousov-Zhabotinsky Reaction"

First Round of Reviewer Comments

Reviewer: 1

Comments to the Author

Referee's Report on

A combined X-ray absorption and UV-Vis spectroscopic study of the iron-catalyzed Belousov-Zhabotinsky reaction by

G. Capocasa *et. al.*

Journal of Physical Chemistry Letters

The allure of the history, significance, demonstrability, and complexity of the Belousov-Zhabotinsky (BZ) reaction has been long-lasting; indeed, it remains a subject of much current interest. This well-written and very welldocumented letter reports analysis of some of the bromine chemistry related to the BZ reaction via the combination of X-ray absorption, UV-Vis spectroscopy and theoretical X-ray absorption when, as observed in the letter, this bromine chemistry is invisible to classical spectroscopy. In addition, some sophisticated modern data processing (using, in particular, principle component analysis) is central to the interpretation of the reported experimental data.

The techniques used in the data analysis are part of the contribution from this research. Also, experimental evidence is provided for the direct transformation of BrO_3^- to bromoallylmalonic acid, which is a central reaction in the oscillatory chemistry. While the experimental techniques are compelling, the interpretation of results is a delicate matter that may require further validation.

Similar ideas have recently been reported (see citation 45) by the same research group (albeit for a slightly different problem) in this journal (vol. 15 (2024) 7312–7319). Thus, the main framework of the experimental techniques has been previously reported.

Except for the existence of the previous letter (citation 45), the submission under review meets the usual standard for a letter in a respected journal.

Reviewer: 2

Comments to the Author

This is a very interesting report on using Br K-edge XANES to measure the bromine speciation in the iron-catalyzed Belousov-Zhabotinsky (BZ) reaction. Despite how famous this reaction is in chemistry demonstrations, the authors make a convincing argument that the complex Br reaction network is still not well understood, due to the fact that it is spectroscopically silent in UV-Vis spectroscopy (at least it was convincing to someone like me who is not well-versed in the literature on this mechanism). Bromine is postulated to exist as Br-, BrO3-, HBrO2, HOBr, BrAMA, and Br2, so the idea of using K-edge XANES to quantify each of these species is a good one. The introduction gives a clear explanation of what is known about the reaction. The major conclusion of this work is that an initial solution primarily consisting of BrO3- is converted on fast then slow timescales to BrAMA with no other observable intermediates, and result is used to put limits on particular reaction rates.

Br K-edge XANES is fairly rare, and the case for publication in JPCL is enhanced by the popularity of the BZ reaction for chemical education. However I have several concerns about the data analysis.

1) The authors perform singular value decomposition of the time-resolved spectra, and conclude based on the scree plot (Figure 3A) that there are only two important components. However, the first three components are perfectly linear, so in my experience it would be more appropriate to consider the third component as well (the plot levels off at component 4). Figure S3 shows that the residual error after two components is about 0.21%, and about 0.1% after three. That's not such a large dropoff that they should ignore the third component (especially because their primary conclusion depends on the fact that there are only 2 components). Normal practice would be to perform the workup using three components, and observe whether there is significant structure in the third component. Only then can they make this conclusion.

2) On that note, the authors should show (in the SI) spectral slices at selected times and the reconstructed spectra from the fit (i.e population1(t=30 sec)*spectrum 1 + population2(t=30 sec)*spectrum2), so that the reader (and reviewer) can judge the quality of the fit. Reconstructed kinetics at important energies would also be useful. They should show these for 2 and 3 components so the reader can judge whether the 3rd component makes a qualitative difference.

3) I am surprised at the very small error of 3% shown in Figure S3A for even a single component. To me that says that if they fit the data with only one component, that the result fits the data within 3%. That clearly can't be right, since the spectrum changes significantly over the experiment. Am I very confused by what this plot is supposed to show? If I am confused here, then further explanation in the SI would be helpful.

4) The authors show that the spectrum of the second component is a good (but not perfect) match to both FDMNES calculations of BrAMA and to the experimental spectrum of the similar

complex diethyl bromomalonate (the latter is in the SI, Figure S4). This is convincing evidence that the second component spectrum is consistent with BrAMA. However, they also need to show that it is NOT consistent with the other postulated species (Br^-, $HBrO_2$, HOBr, and Br_2). One of those species could also be consistent with the spectrum (or be consistent with whatever they get for Component 3 after they look at that). Given the successful use of FDMNES for BrAMA, it should be straightforward to calculate those spectra (or find experimental spectra) and put those in the SI.

Author's Response to Peer Review Comments:

Reviewer: 1

Recommendation: This paper represents a significant new contribution and should be published as is.

Comments:

We thank the Reviewer for his kind appreciation of our work.

Reviewer: 2

Recommendation: This paper may be publishable, but major revision is needed

First of all, we would like to really thank the Reviewer as he has read the manuscript very carefully and he has highlighted some important issues that we considered in the revised version of the manuscript and allowed us to greatly improve it.

Reviewer's comment:

The authors perform singular value decomposition of the time-resolved spectra, and conclude based on the scree plot (Figure 3A) that there are only two important components. However, the first three components are perfectly linear, so in my experience it would be more appropriate to consider the third component as well (the plot levels off at component 4).

Authors' answer:

We thank the Reviewer for highlighting this issue that allowed us to reconsider our analysis and to improve the manuscript. Following the Reviewer's suggestion, we carried out a new analysis including three components. Indeed, a small amount of Br^- is expected to be present in solution at the beginning of the reaction and we imposed the presence of this reagent in the analysis. As a result, a small amount of Br^- has been found to be present that rapidly goes to zero.

In the new version of the manuscript the following sentence has been added at page 4 and Figure 3 has been modified accordingly.

Further, the concentration of Br^- starts from an initial value of ca. 9% (due to the total bromine initially present in the reaction mixture, since [KBr]0 = 8.0 mM) and rapidly decays towards values only slightly above zero, as expected due to the initial minority presence of Br^- in the reaction mixture and to its consumption during the BZ oscillatory cycles

Reviewer's comment:

Figure S3 shows that the residual error after two components is about 0.21%, and about 0.1% after three. That's not such a large dropoff that they should ignore the third component (especially because their primary conclusion depends on the fact that there are only 2 components). Normal practice would be to perform the workup using three components, and observe whether there is significant structure in the third component. Only then can they make this conclusion.

I am surprised at the very small error of 3% shown in Figure S3A for even a single component. To me that says that if they fit the data with only one component, that the result fits the data within 3%. That clearly

can't be right, since the spectrum changes significantly over the experiment. Am I very confused by what this plot is supposed to show? If I am confused here, then further explanation in the SI would be helpful.

Authors' answer:

We thank the Reviewer once more as he spotted a mistake in the calculation of the residual error. We have calculated again the errors for 1, 2 and 3 components using the formula reported in the new version of the Supporting Information as Eq. 2 and a much more reasonable result has been obtained.

We modified Figure S3 and the following sentence has been added at page 4 of the new version of the manuscript:

Consistingly, the percentage residual error committed in reconstructing the XAS dataset with N=3 is negligible, never exceeding a value of 0.006% in the reconstruction of individual XAS spec- tra (Figure S3c), and experimental XAS spec- tra at selected times from reaction start are well

reproduced by employing 3 PCs, as shown in Figure 4.

Reviewer's comment:

On that note, the authors should show (in the SI) spectral slices at selected times and the reconstructed spectra from the fit (i.e population1(t=30 sec)*spectrum 1 + population2(t=30 sec)*spectrum2), so that the reader (and reviewer) can judge the quality of the fit. Reconstructed kinetics at important energies would also be useful. They should show these for 2 and 3 components so the reader can judge whether the 3rd component makes a qualitative difference.

Authors' answer:

As suggested by the Reviewer we reconstructed the experimental XAS spectra at different steps of the reaction using three components and the result is quite convincing. We therefore decided to include this analysis in the main text as Figure 4.

Reviewer's comment:

The authors show that the spectrum of the second component is a good (but not perfect) match to both FDMNES calculations of BrAMA and to the experimental spectrum of the similar complex diethyl bromomalonate (the latter is in the SI, Figure S4). This is convincing evidence that the second component spectrum is consistent with BrAMA. However, they also need to show that it is NOT consistent with the other postulated species (Br-, HBrO2, HOBr, and Br2). One of those species could also be consistent with the spectrum (or be consistent with whatever they get for Component 3 after they look at that). Given the successful use of FDMNES for BrAMA, it should be straightforward to calculate those spectra (or find experimental spectra) and put those in the SI.

Authors' answer:

As suggested by the Reviewer we carried out FDMNES calculations for $HBrO_2$ and HOBr and we show the experimental data for Br^- and Br_2. All these data are reported in Figure S5 that has been added to the SI.

Moreover, the following sentence has been added in the revised version of the manuscript at page 5:

Our assignment is further sup by the fact that the theoretical spectra of $HBrO_2$ and (Figure S5a) and the experimental spectra of Br^- and Br_2 (Figure S5b) display evident differences if compared to the MCR-derived XANES spectrum of the third reaction component.

jz-2024-034903.R2

Name: Peer Review Information for "A Combined X-ray Absorption and UV-Vis Spectroscopic Study of the Iron-Catalyzed Belousov-Zhabotinsky Reaction"

Second Round of Reviewer Comments

Reviewer: 1

Comments to the Author

Good work. Your experimental collaboration seems to be bearing fruit. Continue to refine your experimental and analytic techniques.

Reviewer: 2

Comments to the Author

I am glad that my review was helpful, and the revisions have alleviated all of my concerns. I strongly support this paper being published, with only one small but important change. In Figure 4, the figures showing the residual error need to be zoomed in on the Y axis. When the actual data+fit has a maximum Y axis of between 3 and 1.4 (going from left to right in the figure), it doesn't make sense that the residual plot would have a Y maximum of 1. As plotted, the residual looks like a flat line and gives the reader no opportunity to judge whether there is anything missing in the fit. This axis needs to be zoomed in until the reader can see how much the residual differs from zero, whether that is Y=-0.1 to 0.1 or Y=-0.00001 to 0.00001 . Don't hide the fit error - show it proudly and trust the reader to make a fair judgement.

Author's Response to Peer Review Comments:

Reviewer: 1

Recommendation: This paper is publishable subject to minor revisions noted. Further review is not needed.

<u>Reviewer's comment:</u>

I am glad that my review was helpful, and the revisions have alleviated all of my concerns. I strongly support this paper being published, with only one small but important change. In Figure 4, the

figures showing the residual error need to be zoomed in on the Y axis. When the actual data+fit has a maximum Y axis of between 3 and 1.4 (going from left to right in the figure), it doesn't make sense that the residual plot would have a Y maximum of 1. As plotted, the residual looks like a flat line and gives the reader no opportunity to judge whether there is anything missing in the fit. This axis needs to be zoomed in until the reader can see how much the residual differs from zero, whether that is Y=-0.1 to 0.1 or Y=-0.00001 to 0.00001 . Don't hide the fit error - show it proudly and trust the reader to make a fair judgement.would be more appropriate to consider the third component as well (the plot levels off at component 4).

Authors' answer:

We thank again the Reviewer, and we have modified Figure 4 using a more proper scale for the Y axis as suggested.
